# KMT5A maintains muscle stem cell quiescence through epigenetic regulation of Notch signaling

**DOI:** 10.1016/j.isci.2026.117424

**Published:** 2026-09-04

**Authors:** Jeonghye Hannah Hwang, Adam Y. Chu, Roméo S. Blanc

**Affiliations:** 1 Department of Cell and Regenerative Biology, School of Medicine and Public Health, University of Wisconsin-Madison, Madison, WI, USA

**Keywords:** muscle stem cells, quiescence, activation, notch signaling, KMT5A, SETD8, H4K20 methylation, epigenetics, regeneration

## Abstract

Quiescence preserves muscle stem cell (MuSC) identity and regenerative capacity, but the chromatin mechanisms that support this state remain incompletely understood. Here, we show that the H4K20 monomethyltransferase KMT5A sustains canonical Notch signaling in quiescent MuSCs through catalytic deposition of H4K20me1. Loss of *Kmt5a* led to delayed reduction of *Rbpj* and other canonical Notch pathway genes, whereas acute catalytic inhibition of KMT5A was sufficient to suppress their expression, indicating that H4K20me1 supports transcriptional competence at Notch loci. Genetic restoration of Notch signaling rescued quiescence-associated features in *Kmt5a*-deficient MuSCs but did not restore post-activation survival or regenerative output. During physiological activation after injury, H4K20me1 at *Notch* loci remained largely preserved while H4K20me3 accumulated. Together, these findings identify KMT5A-dependent H4K20me1 as a chromatin mechanism that maintains the Notch-dependent quiescence program and distinguish homeostatic *Kmt5a* loss from physiological MuSC activation.

## Highlights

• KMT5A maintains muscle stem cell quiescence by sustaining Notch signaling
• KMT5A-dependent H4K20me1 preserves transcriptional competence at Notch loci
• Notch restoration rescues quiescence in *Kmt5a*-deficient muscle stem cells
• H4K20me3 increases at H4K20me1-marked Notch loci during physiological activation

## Graphical abstract

**Figure:**
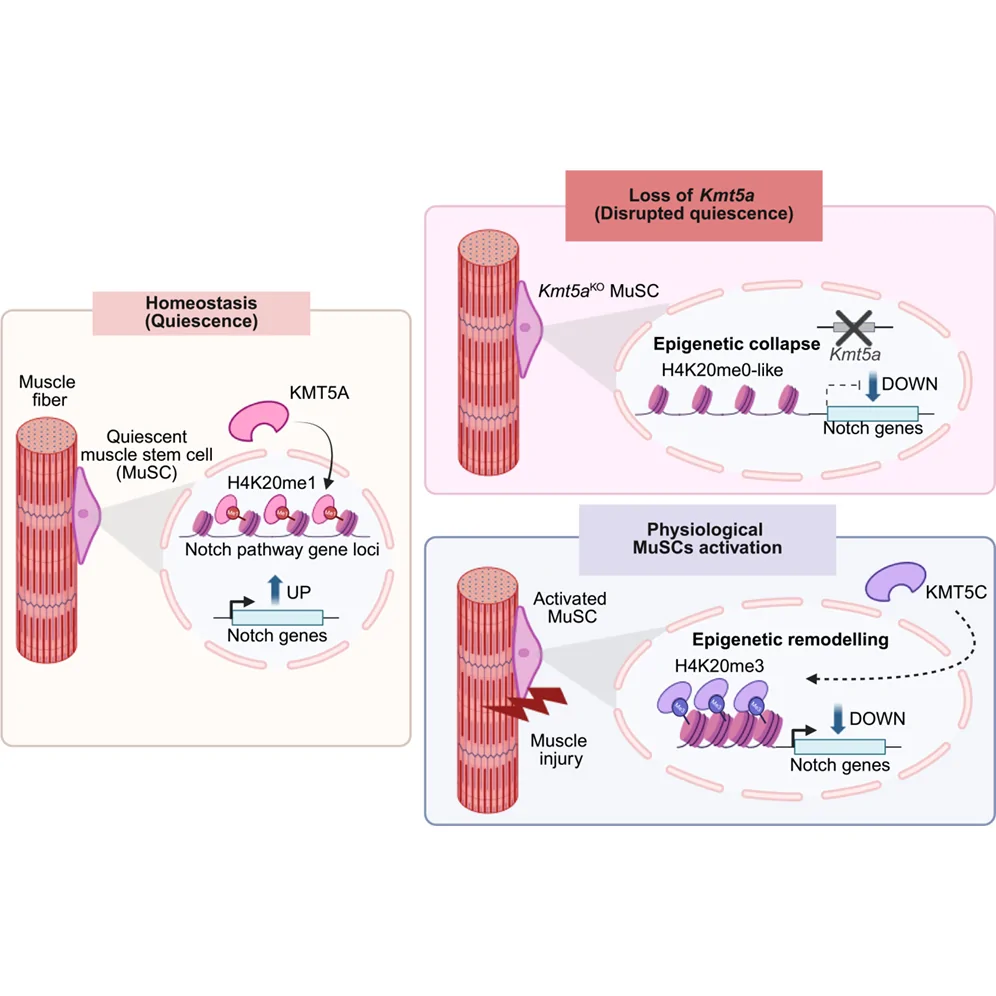

## Teaser

Epigenetics; Developmental biology

## Introduction

Skeletal muscle stem cells (MuSCs), also known as satellite cells, reside in a reversible, non-dividing quiescent state under homeostatic conditions, preserving the stem cell pool and regenerative capacity of skeletal muscle.^1^^,^^2^ Upon injury, MuSCs exit quiescence, activate, proliferate, and generate myogenic progeny to repair damaged fibers, while a subset self-renews and returns to quiescence to replenish the stem cell reservoir.^3^^,^^4^ A tightly regulated balance between quiescence and activation is therefore essential to prevent stem cell exhaustion and ensure lifelong muscle regeneration.

Multiple niche-derived cues and intrinsic pathways converge to maintain MuSC quiescence, with canonical Notch signaling serving as a key regulator.^5^^,^^6^ Activation of Notch receptors releases the Notch intracellular domain (NICD), which associates with RBPJ, the main transcriptional effector of Notch, to drive expression of genes that maintain the stem cell state.^7^ Genetic ablation of *Rbpj* leads to spontaneous MuSC activation and depletion of the stem cell pool,^8^^,^^9^ demonstrating that continuous Notch activity is required to preserve quiescence. However, despite its importance, how chromatin-based mechanisms stabilize Notch signaling during quiescence remains incompletely understood.

Emerging evidence indicates that chromatin regulators cooperate with signaling pathways such as Notch to preserve MuSC quiescence. For example, the Polycomb complex protein, EZH1, preserves quiescence by sustaining Notch signaling through a mechanism independent of its canonical H3K27 methyltransferase activity.^10^ However, the spectrum of chromatin modifiers that support Notch-dependent quiescence and whether histone modifications epigenetically preserve transcriptional competence at canonical Notch pathway loci during MuSC quiescence remain incompletely characterized. KMT5A (SETD8/PR-Set7), the sole enzyme that catalyzes mono-methylation of histone H4 at lysine 20 (H4K20me1), has recently emerged as a critical regulator of MuSC quiescence.^11^^,^^12^ H4K20me1 can serve as a substrate for further methylation to H4K20me2 and H4K20me3, catalyzed by KMT5B and KMT5C, respectively.^13^^,^^14^^,^^15^ While H4K20me1 has been associated with chromatin openness and transcriptional competence of housekeeping genes,^16^ H4K20me3 is enriched at heterochromatic regions and is widely regarded as a repressive histone modification.^14^ We previously demonstrated that the loss of *Kmt5a* in MuSCs triggers a spontaneous, progressive exit from quiescence followed by ferroptotic cell death.^12^ While these findings established KMT5A as essential for stem cell integrity, the mechanism how KMT5A preserves quiescence at the molecular level remained unclear.

Here, using mouse genetics together with transcriptomic and epigenomic profiling, we show that KMT5A sustains canonical Notch signaling in quiescent MuSCs through the catalytic deposition of H4K20me1, thereby preserving stem cell quiescence.

## Results

### KMT5A catalytic activity sustains canonical Notch signaling in quiescent MuSCs

We previously reported that the maintenance of H4K20me1 by the histone methyltransferase KMT5A is essential for preserving MuSC quiescence and survival,^12^ but how KMT5A maintains the quiescent state remained unclear.

**Figure 1:**
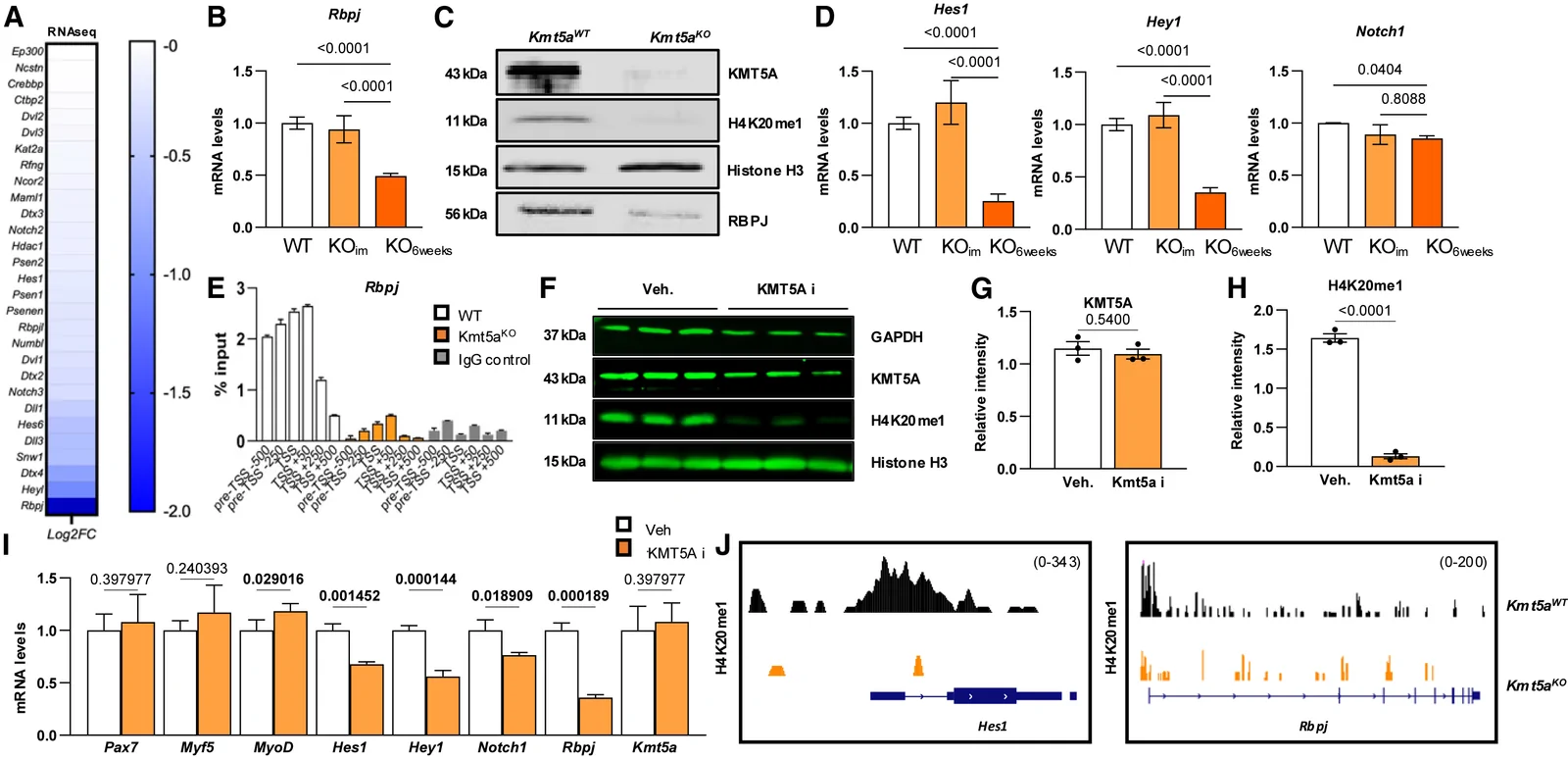
KMT5A catalytic activity sustains canonical Notch signaling in quiescent MuSCs (A) RNA sequencing analysis of MuSCs isolated from WT and inducible *Kmt5a* knockout mice 6 weeks post-deletion (*n* = 3 mice per condition) reveals significant downregulation of canonical Notch signaling genes. Data are displayed as heatmaps of log_2_ fold change. (B) RT-qPCR analysis of *Rbpj* transcript levels 6 weeks after *Kmt5a* deletion in MuSCs. (C) Immunoblot analysis of RBPJ protein levels in MuSCs from KMT5A KO_6weeks_ mice. KMT5A confirms knockout efficiency; H4K20me1 and total H3 serve as modification and loading controls, respectively. (D) RT-qPCR analysis of core Notch pathway components (*Notch1*) and downstream targets (*Hes1* and *Hey1*) 6 weeks post-*Kmt5a* deletion. (E) CUT&RUN-qPCR analysis of KMT5A occupancy at the *Rbpj* transcription start site (TSS) in WT (white), *Kmt5a*-null (orange), and IgG control (gray) MuSCs. (F–H) Immunoblot (F) and quantification of KMT5A (G) and H4K20me1 (H) following 24h treatment with the selective KMT5A catalytic inhibitor UNC0379 (Kmt5a i). Catalytic inhibition reduces H4K20me1 without altering KMT5A protein levels (*n* = 3 independent experiments). (I) RT-qPCR analysis of Notch pathway genes and myogenic differentiation markers following KMT5A inhibitor (KMT5A i) or vehicle (veh) treatment. Data are presented as normalized fold change ± SD (*n* = 3 mice per condition). (J) Representative genome browser tracks of H4K20me1 at Notch pathway loci (*Hes1* and *Rbpj*) demonstrating reduced chromatin occupancy 1 week after *Kmt5a* deletion. Statistical analysis: RT-qPCR and immunoblot quantifications were analyzed using the Welch *t* test (two-tailed) or one-way ANOVA (B and D). Exact *p* values and adjusted *p* values are reported in the figure. Data are presented as mean ± SD.

Reanalysis of our previously published transcriptomic dataset from wild-type (WT) and *Kmt5a* knockout (KO) MuSCs derived from inducible *Kmt5a* MuSC-specific KO mice (Pax7^CreERT2/+^; Kmt5a^fl/fl^ [Kmt5a^KO^])^12^ revealed significant downregulation of canonical Notch pathway genes (Figure 1A). Given the central role of Notch signaling in MuSC quiescence, we asked whether KMT5A acts upstream of the Notch transcriptional program. Among the downregulated genes, *Rbpj* was one of the most affected (Figure 1A). Because RBPJ is the obligate DNA-binding effector of canonical Notch signaling and is required for MuSC quiescence,^8^^,^^9^ we validated its expression in Kmt5a^KO^ MuSCs. Both *Rbpj* mRNA and RBPJ protein levels were reduced following *Kmt5a* deletion (Figures 1B and 1C), indicating that the loss of *Kmt5a* compromises a core component of canonical Notch signaling.

We next asked whether KMT5A loss affects the broader Notch transcriptional program. Expression of representative pathway components and targets, including *Notch1, Hes1*, and *Hey1*, was not immediately reduced after *Kmt5a* deletion but declined progressively and was clearly decreased 6 weeks after deletion (Figures 1B and 1D). This suggests that KMT5A licenses transcriptional competence at Notch loci rather than acting as an acute transcriptional activator. These delayed kinetics are consistent with our previous observation that, despite efficient *Kmt5a* deletion, MuSC quiescence is disrupted progressively rather than synchronously *in vivo*, and MuSC loss occurred progressively and stochastically over several weeks.^12^ Consistent with a direct chromatin association, CUT&RUN-qPCR showed KMT5A enrichment at the *Rbpj* transcription start site in WT MuSCs, and this enrichment was lost in *Kmt5a*-null cells (Figure 1E).

To test whether this regulation is catalytically dependent, and to complement the progressive *in vivo* deletion model, we acutely inhibited KMT5A enzymatic activity with UNC0379.^12^ UNC0379 rapidly reduced H4K20me1 without altering KMT5A protein levels (Figures 1F–1H), thereby enabling acute perturbation of KMT5A catalytic function prior to overt disruption of the MuSC state. Under these conditions, *Rbpj, Hes1, Hey1*, and *Notch1* transcripts were reduced, without evidence of a coordinated myogenic activation/differentiation response, with *Pax7* and *Myf5* remaining unchanged and *MyoD* showing a modest increase (Figure 1I). These findings suggest that impaired Notch pathway expression can occur acutely following loss of KMT5A catalytic activity and is not solely a secondary consequence of long-term quiescence disruption. CUT&Tag analysis further showed strong H4K20me1 enrichment at Notch pathway loci in quiescent MuSCs, which was markedly reduced 1 week after *Kmt5a* deletion (Figure 1J). Together, these data indicate that KMT5A catalytic activity is required to sustain canonical Notch pathway gene expression in quiescent MuSCs through the maintenance of H4K20me1.

### Restoration of Notch signaling rescues quiescence in Kmt5a^KO^ MuSCs

**Figure 2:**
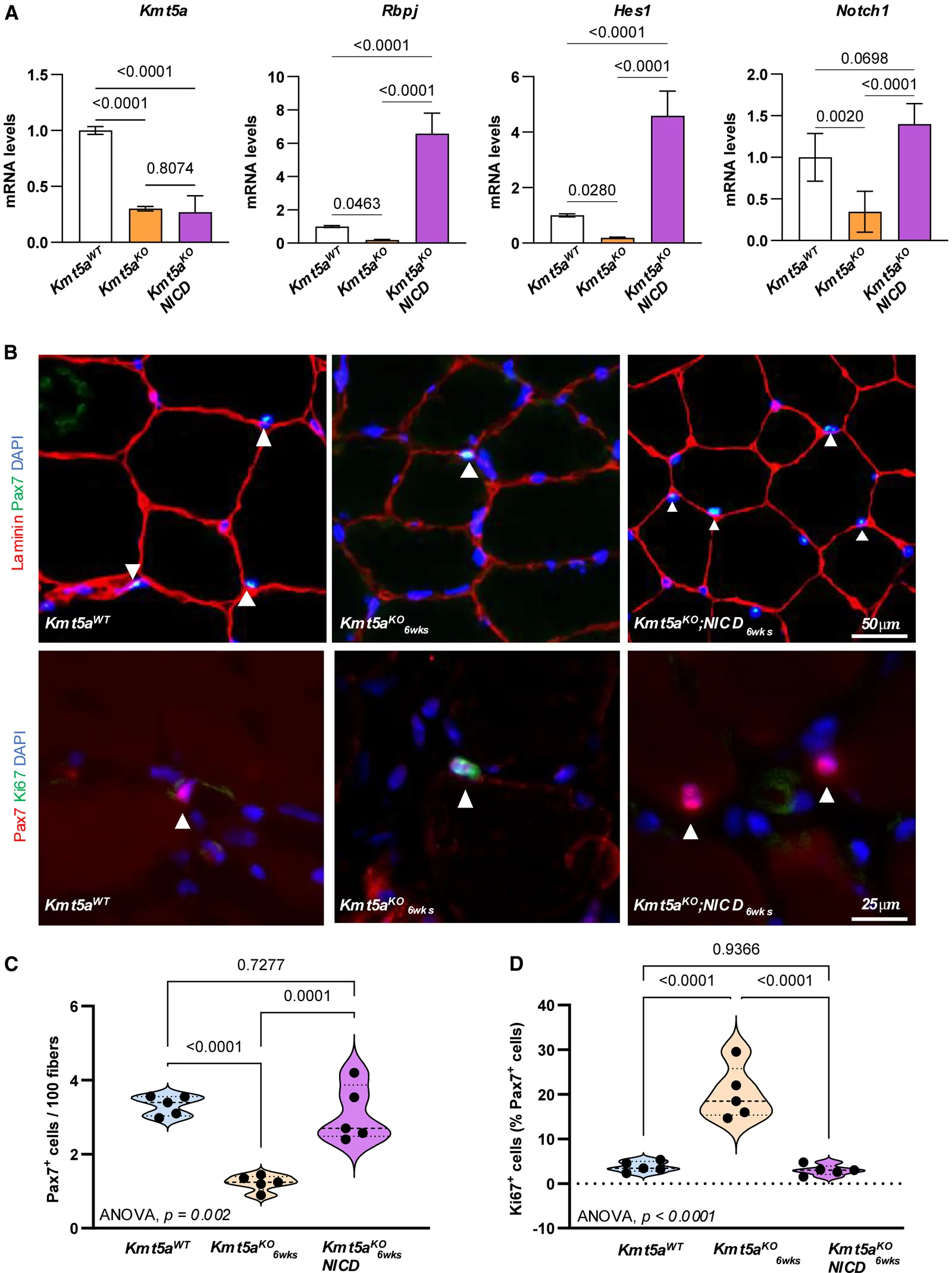
Enforced Notch activation restores quiescence in the absence of KMT5A (A) Pax7^CreERT2/+^; Kmt5a^fl/fl^ mice were crossed with ROSA^NICD^ mice to generate tamoxifen-inducible MuSC-specific double mutants (Kmt5a^KO^;NICD). RT-qPCR analysis confirms restoration of canonical Notch pathway gene expression (*Rbpj*, *Hes1*, *Notch1*) upon NICD overexpression without restoring the expression of *Kmt5a*. qPCR was performed 6 weeks after tamoxifen administration. (B–D) Homeostatic analysis of MuSC quiescence 6 weeks following tamoxifen induction. Immunofluorescence staining for Pax7 and Ki67 was performed to assess total MuSC number (Pax7^+^; white arrow) and proliferating MuSCs (Pax7^+^Ki67^+^). Scale bars, 50 μm (upper) and 25 μm (lower). NICD overexpression prevents depletion of the Pax7^+^ stem cell pool and reduces aberrant activation observed in Kmt5a^KO^ MuSCs. Quantification shown in (C) and (D). Data are presented as mean ± SD unless otherwise indicated. Statistical significance was determined using one-way ANOVA with multiple-comparison correction, as appropriate. Exact *p* values and adjusted *p* values are reported in the figure. See Figure S1 for related content.

Having established that KMT5A sustains Notch signaling in quiescent MuSCs, we next tested whether reduced Notch activity accounts for the quiescence defect in *Kmt5a*-deficient MuSCs. To restore canonical Notch signaling, we crossed Pax7^CreERT2/+^; Kmt5a^fl/fl^ mice with ROSA^NICD^ mice, enabling tamoxifen-inducible NICD expression specifically in MuSCs. Following tamoxifen administration, double-mutant MuSCs showed efficient loss of *Kmt5a* expression comparable with *Kmt5a*-null cells, confirming that NICD overexpression did not restore *Kmt5a* levels (Figure 2A). NICD expression restored *Rbpj*, *Hes1*, and *Notch1* expression in *Kmt5a*-null MuSCs (Figure 2A), consistent with the reactivation of canonical Notch signaling.

Under homeostatic conditions, enforced NICD expression prevented depletion of the Pax7^+^ stem cell pool and reduced the proportion of Ki67^+^ MuSCs in the Kmt5a^KO^ background (Figures 2B–2D). These data indicate that reduced Notch signaling is a major driver of quiescence destabilization in *Kmt5a*-deficient MuSCs and that restoring Notch activity is sufficient to maintain quiescence-associated features under homeostatic conditions.

We next asked whether NICD restoration also rescues post-injury outcomes. At 21 days post-injury, Kmt5a^KO^;NICD muscles showed severe regenerative impairment comparable with Kmt5a^KO^ muscles (Figures S1A–S1C). Both genotypes lacked the regeneration-associated peak of centrally nucleated fiber size seen in WT muscle (Figures S1B and S1C). Likewise, quantification of Pax7^+^ MuSCs at 21 days post-injury (dpi) showed near-complete depletion of the MuSC pool in both Kmt5a^KO^ and Kmt5a^KO^;NICD muscles (Figure S1D). To assess MuSC fate independently of the regenerative environment, freshly isolated MuSCs were plated and monitored *ex vivo*. WT MuSCs remained viable, whereas both Kmt5a^KO^ and Kmt5a^KO;^NICD MuSCs showed a marked decline in survival beginning at 72 h and worsening by 96 h (Figure S1E). Together, these findings indicate that the restoration of Notch activity rescues quiescence-associated features but is not sufficient to restore post-activation survival or regenerative output in the absence of KMT5A.

### Physiological activation of MuSCs is associated with accumulation of H4K20me3 at quiescence-associated Notch loci

**Figure 3:**
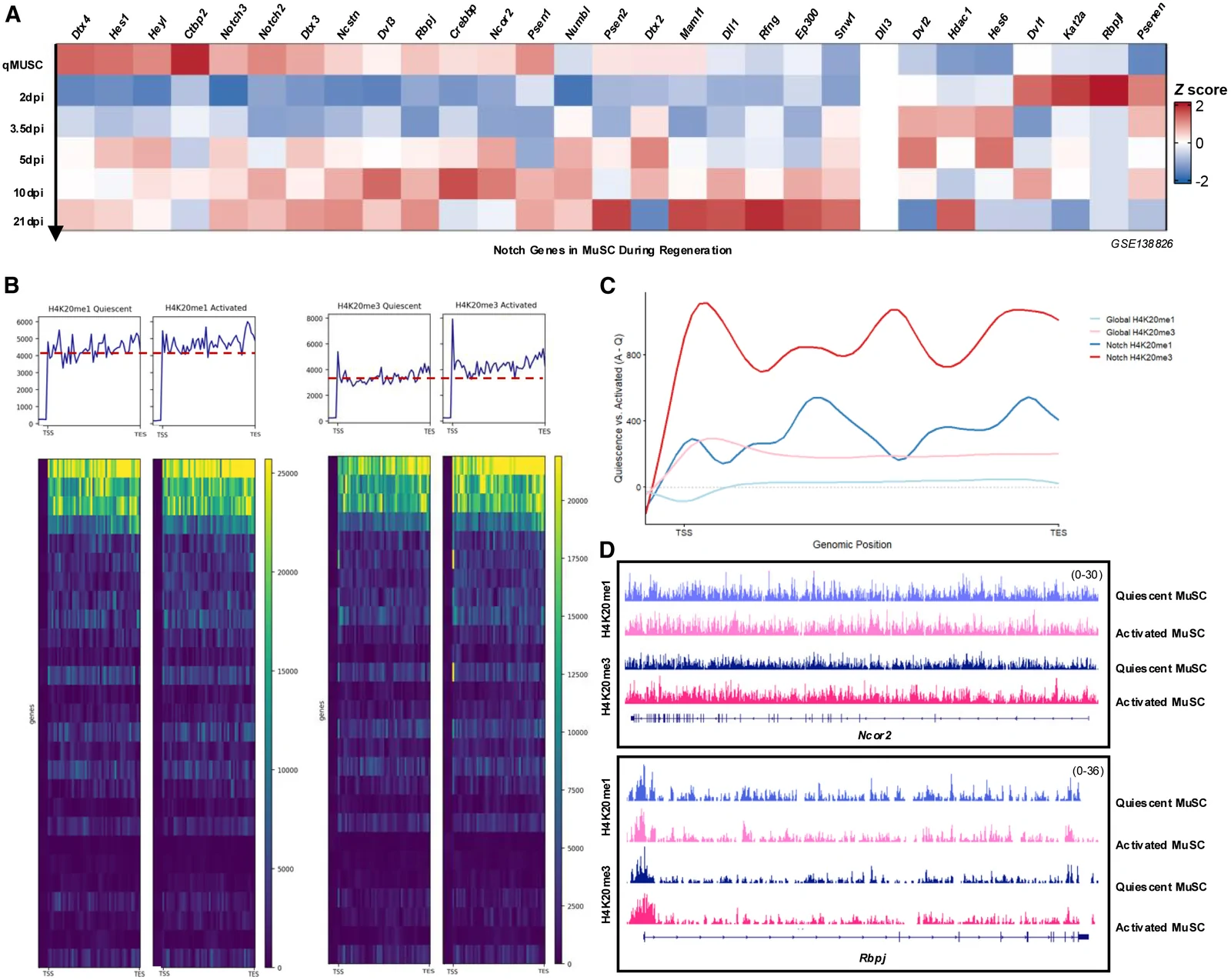
Physiological activation of MuSCs is associated with accumulation of H4K20me3 at Notch signaling loci (A) Heatmap of Notch gene set expression in MuSC during myogenesis. Data were analyzed from publicly available scRNA-seq dataset GEO: GSE138826. (B) Heatmaps and metagene profiles of H4K20me1 and H4K20me3 across the Notch gene set in quiescent and activated MuSCs, ordered by H4K20me3 differential signal. H4K20me1 remains stable, whereas H4K20me3 increases upon activation. MuSCs were activated *in vivo* following BaCl_2_-induced injury and harvested at 2 dpi. (C) Metagene profiles of the difference in CUT&Tag signal between activated and quiescent MuSCs (A − Q) across gene bodies (TSS-TES). Compared with the global profile (light lines), the Notch gene set (dark lines) shows a stronger increase in H4K20me3 upon activation. (D) Representative genome browser tracks of H4K20me1 and H4K20me3 occupancy at the *Ncor2* and *Rbpj* loci in quiescent and activated MuSCs, demonstrating increased H4K20me3 signal upon activation without corresponding loss of H4K20me1.

Given that *Kmt5a* deletion destabilizes quiescence through loss of H4K20me1, we asked whether physiological quiescence exit during injury similarly involves loss of H4K20me1 at Notch-associated loci. To place the chromatin data in transcriptional context, we first mapped Notch pathway gene expression across adult myogenesis using a published single-cell RNA sequencing (scRNA-seq) dataset (Figure 3A).^17^ Quiescence-associated Notch genes, including *Hes1*, *Heyl*, and *Rbpj*, were highest in quiescent MuSCs, reduced at 2 dpi, and re-expressed as regeneration progressed (Figure 3A), consistent with prior work.^8^^,^^18^ Unlike the canonical Notch effector *Rbpj*, the paralog *Rbpjl*, which has been reported to be non-responsive to canonical Notch activation, displayed distinct expression dynamics in these datasets.^19^

We then performed CUT&Tag profiling for H4K20me1 and H4K20me3 in freshly isolated quiescent MuSCs and MuSCs activated *in vivo* 2 days after BaCl_2_ injury. Because Notch pathway genes are reduced during MuSC activation (Figure 3A) and after *Kmt5a* deletion (Figure 1), we initially expected H4K20me1 at these loci to decrease. Instead, H4K20me1 across the Notch gene set remained largely unchanged in activated MuSCs, whereas H4K20me3 increased markedly (Figures 3B–3D). Because H4K20me1 serves as the substrate for further methylation to H4K20me3, these data are consistent with progression toward H4K20 tri-methylation at Notch loci during physiological activation. Metagene analysis further showed a stronger increase in H4K20me3 across the Notch gene set than across the global gene profile (Figure 3C). Thus, physiological activation is not associated with loss of H4K20me1 at Notch loci, but rather with accumulation of H4K20me3, consistent with a more repressive chromatin state at these genes as MuSCs exit quiescence, although the functional significance of this accumulation remains to be determined.

**Figure 4:**
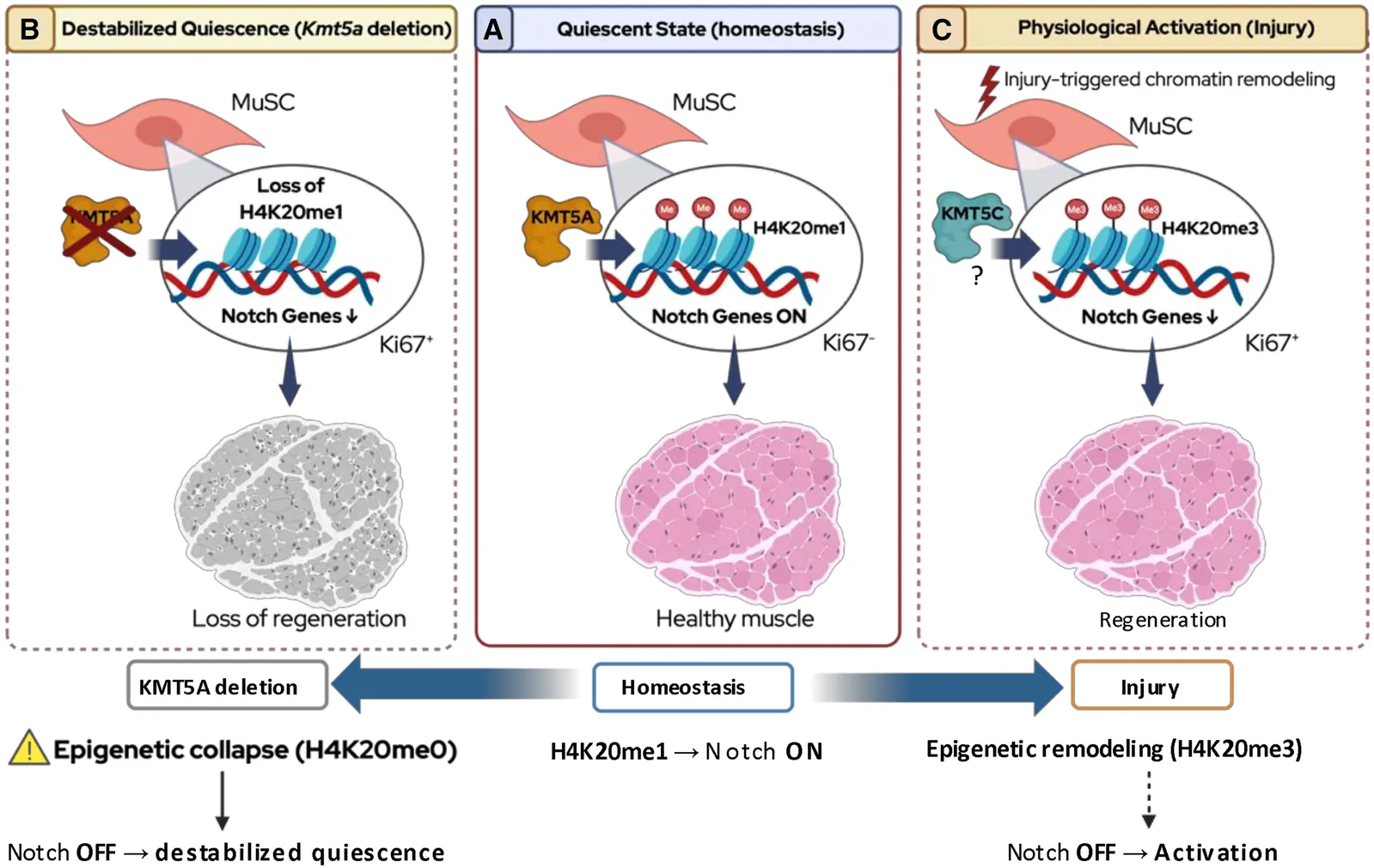
Model of phase-specific H4K20 methylation dynamics during MuSC state transitions In homeostatic MuSCs, KMT5A-dependent H4K20me1 maintains Notch transcription and quiescence (A). *Kmt5a* deletion leads to loss of H4K20me1 without compensatory H4K20me3, resulting in epigenetic collapse and destabilized quiescence (B). In contrast, injury-driven activation is characterized by H4K20me3 accumulation at Notch loci, reflecting regulated chromatin remodeling rather than loss of maintenance (C). Created with BioRender.

These results distinguish the chromatin changes accompanying physiological activation from those triggered by *Kmt5a* deletion during homeostasis. In particular, injury-driven quiescence exit is associated with H4K20me3 accumulation at Notch loci rather than H4K20me1 depletion, indicating that genetic destabilization of quiescence and physiological activation may be accompanied by distinct H4K20 methylation dynamics (Figure 4).

## Discussion

Maintenance of MuSC quiescence requires active reinforcement of dormancy-enforcing pathways while preserving responsiveness to regenerative cues. Canonical Notch signaling is central to this balance, and the disruption of Notch-RBPJ signaling destabilizes adult MuSC quiescence and depletes the stem cell pool.^8^^,^^9^ Niche-level circuits such as the Notch-COLV-CALCR axis further underscore that Notch signaling is not merely permissive but also an active stabilizer of MuSC quiescence state *in vivo.*^20^ Recent work also emphasizes that Notch output is highly context dependent across myogenic stages and cell states, including stage-specific functions of Dll1 and distinct effects of Notch activation during later phases of myogenesis.^21^^,^^22^^,^^23^^,^^24^ Within this framework, our study identifies KMT5A-dependent H4K20me1 as an epigenetic mechanism that supports the quiescent Notch program.

Our findings add to emerging evidence that multiple, biochemically distinct chromatin regulators converge on the canonical Notch signaling to preserve MuSC quiescence through mechanistically distinct pathways. EZH1, the catalytically attenuated Polycomb homolog, similarly sustains Notch signaling in quiescent MuSCs, yet does so through a mechanism independent of its H3K27 methyltransferase activity.^10^ In contrast, the present study identifies a direct histone methylation-based mechanism in which KMT5A catalytic activity maintains H4K20me1 at canonical Notch pathway loci, thereby preserving their transcriptional competence in quiescent MuSCs. Together, these studies suggest that Notch-dependent quiescence is actively maintained by at least two parallel epigenetic layers. Whether KMT5A-dependent H4K20me1 and EZH1 regulate overlapping or distinct Notch target loci, and whether these mechanisms operate independently or coordinately, will be important questions for future work.

The loss of *Kmt5a* led to progressive reduction of *Rbpj* and canonical Notch pathway genes. Acute catalytic inhibition of KMT5A phenocopied this transcriptional effect, consistent with a role for H4K20me1 in maintaining transcriptional competence rather than acutely activating transcription. Importantly, the delayed kinetics observed in the inducible *Kmt5a* deletion model are likely biologically informative rather than a limitation of the system. Although H4K20me1 at Notch-associated loci decreased rapidly after *Kmt5a* deletion, reductions in Notch pathway transcripts became more pronounced over time. This is consistent with the proposed role of KMT5A as an epigenetic maintenance factor rather than an acute transcriptional activator. In inducible adult KO systems, residual KMT5A protein and pre-existing chromatin states may transiently preserve transcriptional competence even after efficient genetic recombination. Moreover, our prior work showed that H4K20me1 erosion and MuSC loss following *Kmt5a* deletion occur progressively rather than uniformly or immediately.^12^ Together, these observations support a model in which KMT5A-dependent H4K20me1 stabilizes a chromatin environment permissive for canonical Notch pathway expression, with progressive destabilization of this state leading to delayed transcriptional decline. Nevertheless, because the chronic *Kmt5a* deletion model undergoes progressive disruption of MuSC quiescence over time, we cannot fully exclude the possibility that part of the observed Notch transcriptional reduction reflects secondary reinforcement of MuSC state changes rather than exclusively primary chromatin-mediated regulation at Notch loci.

Genetic restoration of NICD corrected quiescence-associated features under homeostatic conditions, indicating that impaired Notch signaling is a major driver of the premature activation phenotype in *Kmt5a*-deficient MuSCs. Enforced NICD expression also partially restored *Rbpj* transcript levels in *Kmt5a*-null cells. Positive-feedback architectures within the Notch pathway have been described in other contexts, and such a mechanism could account for this observation^24^; its basis in MuSCs was not further investigated here. At the same time, NICD did not restore post-activation survival or regenerative output. Rather than representing a limitation of the model, the incomplete rescue by NICD restoration is consistent with the broader functions of KMT5A in MuSC biology. Previous work from our group demonstrated that KMT5A is required not only for maintenance of MuSC quiescence, but also for protection against ferroptotic cell death following activation.^12^ Thus, while restoration of NICD is sufficient to rescue quiescence-associated features under homeostatic conditions, it is not unexpected that NICD expression fails to restore post-activation survival or regenerative capacity in the absence of KMT5A. Importantly, although prior studies established that KMT5A is essential for MuSC homeostasis and showed that *Kmt5a* loss disrupts quiescence, the molecular mechanism underlying its role in quiescence maintenance remained unclear. The present study addresses this gap by identifying a KMT5A-H4K20me1 axis that preserves transcriptional competence of canonical Notch pathway genes as a key chromatin-based mechanism supporting MuSC quiescence. Together, these findings suggest that KMT5A stabilizes the MuSC state through both a Notch-dependent quiescence module and additional pathways required for long-term stem cell fitness after activation.

Because *Kmt5a* deletion was restricted to the MuSC compartment using Pax7CreERT2, the molecular and *ex vivo* phenotypes observed here strongly support a MuSC-intrinsic role for KMT5A. Nevertheless, the regenerative defects observed *in vivo* may reflect the combined consequences of intrinsic MuSC dysfunction and interactions with the regenerating tissue environment. This aligns with prior work showing that Notch ligands can promote a stem-like state *ex vivo* without fully preserving regenerative capacity, reinforcing the idea that maintenance of Notch-associated identity and long-term regenerative competence are related but not identical properties.^24^^,^^25^

This study is also consistent with prior studies linking H4K20-dependent chromatin organization to MuSC quiescence. Suv4-20h1-dependent facultative heterochromatin constrains MuSC activation and preserves long-term regenerative capacity.^26^ Thus, distinct steps within the H4K20 methylation pathway appear to support MuSC homeostasis through non-equivalent chromatin mechanisms. Higher H4K20 methylation states can reinforce heterochromatin-associated configurations, whereas H4K20me1 can support transcriptional competence at selected loci, including Notch genes.

The most unexpected observation in our study was that physiological activation did not simply mirror *Kmt5a* deletion. Although *Kmt5a* loss reduced H4K20me1 at Notch loci and destabilized quiescence, injury-induced activation was not accompanied by a comparable depletion of H4K20me1. Instead, H4K20me3 increased across the Notch gene set. In other systems, H4K20me3 accumulation is associated with transcriptional silencing,^27^ making it plausible that MuSC activation entails progression toward a more repressive H4K20 state at quiescence-associated loci. Importantly, however, our data directly demonstrate the accumulation of H4K20me3 at specific loci. We therefore propose that homeostatic *Kmt5a* deletion and physiological activation occupy distinct chromatin states, rather than representing simple variants of the same transition.

This distinction may help explain why *Kmt5a* loss does not produce a productive regenerative transition. In our previous work, the loss of H4K20me1 mediated by pathological conditions caused spontaneous MuSC activation followed by ferroptotic loss.^12^ This study now shows that the early quiescence defect is mediated in large part through failure to sustain canonical Notch signaling. Together, these findings suggest that KMT5A-dependent H4K20me1 preserves MuSC homeostasis at multiple levels, by maintaining the Notch-dependent quiescence program in resting cells and by supporting additional functions required for survival after activation. Whether H4K20me3 deposition is functionally required for efficient repression of quiescence-associated genes during activation, how selective these H4K20 dynamics are across the genome, and how they intersect with other niche- and chromatin-based regulators of Notch signaling will be important questions for future work.

### Limitations of the study

While physiological activation was associated with increased H4K20me3 accumulation at Notch-associated loci, our data do not demonstrate that H4K20me3 deposition is functionally required for Notch repression or activation. Future studies will be required to define the functional significance of this transition.

H4K20me2 dynamics were not directly examined. Although H4K20me2 is generated downstream of H4K20me1 and is considered relatively stable in MuSCs, whether locus-specific H4K20me2 dynamics contribute to transcriptional regulation downstream of KMT5A remains an interesting question for future investigation.

In addition, all *in vivo* experiments in this study were performed using male mice, which may limit the generalizability of our findings across sexes. Future studies including both male and female mice will be important to determine whether KMT5A-mediated regulation of Notch signaling and MuSC quiescence is conserved between sexes.

## Resource availability

### Lead contact

Requests for further information and resources should be directed to and will be fulfilled by the lead contact, Roméo S. Blanc (rblanc@wisc.edu).

### Materials availability

This paper does not report new unique reagents.

### Data and code availability

• CUT&Tag sequencing data generated in this study have been deposited at Gene Expression Omnibus (GEO) and are publicly available. Accession numbers are listed in the key resources table. This paper also analyzes existing, publicly available datasets deposited at GEO; accession numbers are listed in the key resources table.
• This paper does not report original code.
• Any additional information required to reanalyze the data reported in this paper is available from the lead contact upon request.

## Acknowledgments

This work was supported in part by startup funding provided by the University of Wisconsin-Madison (UW-Madison) to R.S.B., including support from the Department of Cell and Regenerative Biology, the Office of the Vice Chancellor for Research (UW-Madison), the 10.13039/100007923UW Carbone Cancer Center, and the UW-Madison Stem Cell and Regenerative Medicine Center, and the 10.13039/100000049National Institute on Aging (K99/R00AG071736, R.S.B.). We thank the Department of Cell and Regenerative Biology Summer Undergraduate Internship Program for financial support to A.Y.C. We also thank Audrey Kopp and Dr. Marjorie Brand for generously sharing their CUT&Tag expertise and reagents.

## Author contributions

Conceptualization, R.S.B.; methodology, J.H.H., A.Y.C., and R.S.B.; investigation, J.H.H. and R.S.B.; writing – original draft, J.H.H. and R.S.B.; writing – review and editing, J.H.H., A.Y.C., and R.S.B.; funding acquisition, R.S.B.; resources, R.S.B.; supervision, R.S.B.

## Declaration of interests

The authors declare no conflict of interests.

## Declaration of generative AI and AI-assisted technologies in the writing process

During the preparation of this work, the authors used ChatGPT (OpenAI) in order to assist with language refinement and manuscript editing. After using this tool or service, the authors reviewed and edited the content as needed and take full responsibility for the content of the publication, which reflects their original scientific work. The AI tool was not used for data analysis, interpretation, or generation of original scientific content.

## STAR★Methods

### Key resources table

**Table:** 

REAGENT or RESOURCE	SOURCE	IDENTIFIER
**Antibodies**		

Rabbit monoclonal anti-H4K20me1	Abcam	Ab177188; RRID: AB_3674578
Rabbit polyclonal anti-H4K20me3	Abcam	ab9053; RRID: AB_306969
Guinea pig anti-rabbit IgG (H + L) secondary	Novus Biologicals	NBP1-72763; RRID: AB_11024108
Mouse monoclonal anti-Pax7	Developmen-tal Studies Hybridoma Bank (DSHB)	RRID: AB_528428
Rabbit anti-KMT5A/SETD8	Milli-pore	06–1304; RRID: AB_1977488
Rabbit anti-KMT5A/SETD8	Cell Signaling Technology	2996; RRID: AB_2254384
Rabbit polyclonal anti-Laminin (LAMA1)	Sigma-Aldrich	L9393; RRID: AB_477163
Rabbit anti-Ki67 conjugated to Alexa Fluor 488	Cell Signaling Technology	11882; RRID: AB_2687824
Rabbit polyclonal anti-H3K4me3	Abcam	ab8580; RRID: AB_306649
Mouse monoclonal GAPDH	Invitrogen	AM4300; RRID: AB_437392
Mouse monoclonal anti-RBPJ	Santa Cruz	sc-271128; RRID: AB_10610612
Rabbit polyclonal anti-Histone H3	Abcam	Ab1791; RRID: AB_302613
IgG Negative Control Antibody	Epicypher	13–0042; RRID: AB_2923178
Rabbit monoclonal anti-H3K27me3	Cell Signaling Technology	9733S; RRID: AB_2616029
Goat anti-Rabbit IgG (H + L) Secondary Antibody, DyLight™ 488	Invitrogen	35552; RRID: AB_844398
Goat anti-Mouse IgG (H + L) Secondary Antibody, DyLight™ 488	Invitrogen	35502; RRID: AB_844397

**Chemicals,peptides,andrecombinantproteins**		

UNC0379 (KMT5A catalytic inhibitor)	MedChemExpress	HY-12335
Barium chloride (BaCl_2_) dihydrate	Sigma-Aldrich	B0750
Collagenase Type II	Gibco	17101–015
Dispase	Gibco	17105–041
BioMag®Plus Concanavalin A	Bangs Laboratories	BP531
pA-Tn5 fusion protein pre-loaded with MEDS adapters	Li et al.^28^ (Dr. Marjorie Brand lab, UW-Madison)	N/A
Digitonin	Sigma-Aldrich	300410
Spermidine	Sigma-Aldrich	S2626
TRIzol Reagent	Invitrogen	15596026
SsoFast™ EvaGreen® Supermix	Bio-Rad	1725202
ECL reagent	Bio-Rad	1705061
EDTA-free cOmplete Protease Inhibitor Cocktail	Roche	04693132001
PhosSTOP Phosphatase Inhibitor Cocktail	Roche	4906845001
Geltrex™ LDEV-Free Reduced Growth Factor Basement Membrane Matrix (ECM)	Gibco	A14132-02
Fluoromount-G mounting medium	SouthernBiotech	0100–01
Phenol-chloroform	Fisher BioReagents™	BP1752I-400
Isoflurane	Dechra	N/A
Tamoxifen	Sigma-Aldrich	T5648
Tissue-Tek O.C.T. Compound	SAKURA	4583
Mouse-on-Mouse IgG blocking solution	Invitrogen	R37621
Proteinase K	Invitrogen	25530–015
TCM® defined serum replacement	MP Biomedicals	2010026
Normal Goat Serum	Jackson ImmunoResearch	005–000–121

**Criticalcommercialassays**		

Satellite Cell Isolation Kit, mouse	Miltenyi Biotec	130–104–268
Anti-Integrin alpha-7 MicroBeads, mouse	Miltenyi Biotec	130–104–261
CUTANA CUT&RUN Kit	Epicypher	14–1048
Qubit 1× dsDNA High Sensitivity (HS) Assay Kit	Invitrogen	Q33230
PicoGreen	Invitrogen	P7581
NEBNext® High-Fidelity 2× PCR Master Mix	New England Biosystems	M0541S

**Depositeddata**		

CUT&Tag	This paper	GEO: GSE324960
Bulk RNA-seq	Blanc et al.^12^	GEO: GSE228013
scRNA-seq	Oprescu et al.^17^	GEO: GSE138826
scRNA-seq	Blanc et al.^12^	GEO: GSE228055
mm10 reference genome	UCSC Genome Browser	https://hgdownload.soe.ucsc.edu/goldenPath/mm10/bigZips/
ENCODE DAC blacklist ENCFF547MET	Amemiya et al.^29^	ENCODE: ENCFF547MET

**Experimentalmodels:Organisms/strains**		

Mouse: C57BL/6J	Jackson Laboratory	000664; RRID: IMSR_JAX:000664
Mouse: Pax7^CreERT2^	Jackson Laboratory	017763; RRID: IMSR_JAX:017763
Mouse: Kmt5a^fl/fl^	Myers et al.^30^	N/A
Mouse: Kmt5a^fl/fl^; Pax7^CreERT2/+^ (Kmt5a^KO^)	This paper	N/A
Mouse: ROSA^NICD^	Pan et al.^31^	N/A
Mouse: Kmt5a^fl/fl^; ROSA^NICD^; Pax7^CreERT2^	This paper	N/A

**Oligonucleotides**		

Primers for qRT-PCR	This paper	Table S1
Primer: Universal i5 and i7 primers	Li et al.^28^	N/A

**Software andalgorithms**		

Trim Galore (v0.6.10-1)	Kreuger	https://github.com/FelixKrueger/TrimGalore
Bowtie2 (v2.5.4)	Langmead et al.^32^	https://github.com/benlangmead/bowtie2
Samtools (v1.22.1)	Danecek et al.^33^	https://github.com/samtools/samtools
Picard (v3.4.0)	Broad Institute	https://github.com/broadinstitute/picard
bedtools (v2.31.1)	Quinlan and Hall^34^	https://github.com/arq5x/bedtools2
deepTools (v3.5.6)	Ramírez et al.^35^	https://github.com/deeptools/deeptools
IGV (Integrative Genomics Viewer)	Robinson et al.^36^	https://github.com/igvteam/igv
Seurat (v5.4.0)	Hao et al.^37^	https://github.com/satijalab/seurat
ComplexHeatmap R package	Gu et al.^38^	https://github.com/jokergoo/ComplexHeatmap
GraphPad Prism (v9 or v10)	GraphPad Software	https://www.graphpad.com/
R (v4.x)	R Core Team	https://www.r-project.org/
ImageJ2/FIJI (v1.52, 1.53, 1.54p)	Reuden et al.^39^ Schindelin et al.^40^	https://fiji.sc/
Echo PRO software (v4.2)	Echo	https://discover-echo.com/
LI-COR Image Studio software	Li-COR Biotech	https://www.licorbio.com/image-studio

**Other**		

Echo Revolve fluorescence microscope	ECHO	Revolve2-K4-2259
LI-COR Odyssey Fc Imager	LI-COR Biosciences	N/A
7500 Fast Real-Time PCR System	Applied Biosystems	4351106
gentleMACS Octo Tissue Dissociator with heaters	Miltenyi Biotec	130–134–029
MultiMACS Cell24 Separator Plus	Miltenyi Biotec	130–098–637
DynaMag™-2 Magnet	Invitrogen	12321D

### Experimental model and study participant details

#### Mice

All animal experiments in this study were carried out in accordance with guidelines set by the Institutional Animal Care and Use Committee (IACUC) at the University of Rochester (Protocol 2013-002e; ref. 102192) and at the University of Wisconsin-Madison (Protocol M006850). Protocol oversight and animal care compliance were provided by the Division of Comparative Medicine (URMC) and the Research Animal Resources and Compliance Unit (UW-Madison). Biosafety protocols were reviewed and approved by the Institutional Biosafety Committee at the URMC and the Office of Biological Safety at UW-Madison. This research was conducted in accordance with guidelines set forth by the National Institutes of Health and the National Institute on Aging, where applicable.

Male C57BL/6J adult mice (4–6 months; #000664; Jackson Laboratory) were used for experiments. Pax7^CreERT2/+^; Kmt5a^fl/fl^ [Kmt5a^KO^] were generated by crossing Kmt5a^fl/fl^ (gifted by Dr. Laurie Steiner, URMC)^30^ and Pax7^CreERT2^ (#017763; Jackson Laboratory). Kmt5a^fl/fl^; ROSA^NICD^; Pax7^CreERT2^ were generated by crossing ROSA^NICD^ Notch gain-of-function allele generously gifted by Dr. Kiernan (URMC)^31^^,^^41^ and our Pax7^CreERT2/+^; Kmt5a^fl/fl^. For experiments comparing wild-type (WT) and Kmt5a^KO^ conditions, Cre-negative littermates (Kmt5a^fl/fl^, no Cre) served as WT controls. Conditional knockout was induced by tamoxifen (T5648; Sigma-Aldrich) administration for 5 consecutive days. KO_im_ refers to mice harvested the day after the final tamoxifen injection, whereas KO_6weeks_ refers to mice harvested 6 weeks after the tamoxifen administration.

For regeneration assays and activating MuSCs, mice were briefly anesthetized with isoflurane (Dechra) and tibialis anterior (TA) muscles were injected with 50-80μL of a 1.2% solution of BaCl_2_ (B0750; Sigma-Aldrich) in normal saline.

#### Primary muscle stem cell isolation and culture

For MuSC isolation, hindlimb skeletal muscles were freshly isolated and dissociated using gentleMACS Octo Tissue Dissociator with heaters (130–134–029; Miltenyi Biotec) in Collagenase II (17101-015; Gibco) and Dispase (17105-041; Gibco) solution, and subjected to magnetic activated cell sorting (MACS) (130–098–637; Miltenyi Biotec) using a Satellite Cell Isolation Kit (130–104–268; Miltenyi Biotec), and Anti-Integrin α-7 MicroBeads (130–104–261; Miltenyi Biotec) as previously described.^42^

Following isolation, freshly isolated MuSCs were processed for direct molecular analysis except for KMT5A inhibitor treatment, in which cells were cultured for 24h. For CUT&Tag analysis, non-injured, freshly isolated MuSCs are considered quiescent, whereas MuSCs harvested 2 days post-injury (2 dpi) are referred to as activated MuSCs.

For KMT5A catalytic inhibition, freshly isolated MuSCs were plated on 0.5% ECM (A14132-02; Gibco) in growing media (DMEM-F12, 20% FBS, 2% TCM defined serum replacement (2010026; MP Biomedicals), 1% pen/strep, 1% HEPES), and were incubated at 37 °C in humidified 5% CO_2_ conditions in the presence of the KMT5A catalytic inhibitor UNC0379 (HY-12335; MedChemExpress) (4 μM) or vehicle control (DMSO). Cells were harvested for immunoblotting and RT-qPCR 24 h after treatment.

### Method details

#### Muscle section and immunostaining

Skeletal muscles were harvested, incubated in 30% sucrose overnight, and frozen with O.C.T. compound (4583; SAKURA) in isopentane cooled in liquid nitrogen and stored at −80 °C prior to sectioning. Frozen muscles were sectioned at 7–10 μm. Muscle sections were fixed for 10 min in 10% normal buffered formalin and permeabilized with 2% NGS/PBS-T (0.2% Triton X-100) for 30 min at room temperature and blocked in 10% NGS (005–000–121; Jackson ImmunoResearch) in PBS-T for 30 min at room temperature. When mouse primary antibodies were used, sections were additionally blocked with Mouse-on-Mouse (R37621; Invitrogen) at room temperature for 1 h. Primary antibody incubation in 5% NGS/PBS-T was carried out at 4 °C overnight. After washes, sections were incubated with secondary antibodies in 5% NGS/PBS-T for 1 h at room temperature. DAPI staining was used to label nuclei. All slides were mounted with Fluoromount-G (0100-01; SouthernBiotech). At least three sections from three slides were analyzed per sample. Sections and cells were imaged on an Echo Revolve Microscope through Echo PRO software (version 4.2) and processed and analyzed in ImageJ2 (versions 1.52, 1.53 and 1.54p).^39^ Sample analysis was conducted in a double-blind manner.

#### Immunoblotting

Cell lysates (50 mM HEPES [pH 7.4], 150 mM NaCl, and 1% Triton X-100) supplemented with freshly added EDTA-free protease inhibitor (04693132001; Roche) and phosphatase inhibitor cocktails (4906845001; Roche) were immunoblotted with primary antibodies overnight at 4C.

KMT5A (06–1304; Milli-pore or 2996; Cell Signaling Technology), H4K20me1 (Ab177188; Abcam), Histone H3 (Ab1791; Abcam), RBPJ (sc-271128; Santa Cruz), GAPDH (AM4300; Invitrogen) primary antibodies were used. HRP-conjugated secondaries and ECL (1705061; Bio-Rad) was used for chemiluminescence detection, while Dylight secondary antibodies (35552, 35502; Invitrogen) were used for infrared detection on LI-COR Odyssey Fc Imager (LI-COR Biosciences).

#### CUT&Tag

CUT&Tag was performed largely as previously described,^28^^,^^43^ with minor modifications. Concanavalin A magnetic beads (BP531; Bangs Laboratories) were activated by washing twice in binding buffer [20 mM HEPES (pH 7.5), 10 mM KCl, 1 mM CaCl_2_, 1 mM MnCl_2_, and 0.1% bovine serum albumin (BSA)] and resuspended in 10ul of binding buffer per sample. Freshly isolated MuSCs were washed with 1 mL of wash buffer [20 mM HEPES (pH 7.5), 150 mM NaCl, 0.83 mM spermidine (S2626; Sigma-Aldrich), 0.1% BSA, and one Roche cOmplete Protease Inhibitor EDTA-free tablet (04693132001; Roche)] and resuspended in 1 mL of wash buffer. 150,000 MuSCs were used per CUT&Tag reaction. Activated bead suspension was added to the cells and the mixture was rotated end-over-end for 20 min at room temperature to allow bead–cell binding. After a brief spin, the tubes were placed on a magnetic rack (12321D, Invitrogen), and the wash buffer was replaced with 150 μL of antibody buffer per sample [wash buffer and digitonin (0.5 μg/mL) (300410; Sigma-Aldrich) and 2 mM EDTA]. 6 μL of rabbit anti-H4K20me1 (ab177188; Abcam), or 6 μL of rabbit anti-H4K20me3 (ab9053; Abcam) antibodies were added to each tube. Samples were incubated overnight at 4°C on an end-over-end rotator. After a brief spin, the tubes were placed on a magnetic rack, and the supernatant was replaced with 150 μL of digitonin-wash buffer [wash buffer with digitonin (0.5 μg/mL)] containing guinea pig anti-rabbit IgG (H + L) secondary antibody (NBP1-72763; Novus Biologicals) at a 1:100 dilution. After an hour of incubation at room temperature on an end-over-end rotator, cells were washed three times with digitonin-wash buffer. The pA-Tn5 (Gifted by Dr. Marjorie Brand, UW-Madison)^28^ solution was prepared by diluting the enzyme 1:250 in digitonin-300 wash buffer [20 mM HEPES (pH 7.5), 300 mM NaCl, 0.83 mM spermidine, digitonin (0.1 μg/mL), 0.1% BSA, and one Roche cOmplete Protease Inhibitor EDTA-free tablet], and 150 μL was added to each sample. pA-Tn5 used in CUT&Tag was preloaded with MEDS adapters. After incubating at room temperature for an hour on an end-over-end rotator, cells were washed three times with digitonin-300 wash buffer. After a quick spin, supernatant was replaced with 300 μL of tagmentation buffer [digitonin-300 wash buffer and 10 mM MgCl_2_] and incubated rotating at 37°C for an hour for tagmentation. 10 μL of 0.5 M EDTA was added to stop tagmentation, followed by addition of 3 μL of 10% SDS and 3 μL of proteinase K (20 mg/mL: 25530015; Invitrogen). Cells were incubated at 37°C overnight on a thermomixer at 600 rpm. DNA was purified by phenol-chloroform (BP1752I-400; Fisher BioReagents) extraction and ethanol precipitation. DNA libraries were amplified by PCR with indexed i5 and i7 primers; the number of cycles was determined by qPCR. Sample concentration was determined using Qubit 1× dsDNA HS Assay (Q33230, Invitrogen). The quality of the library was assessed using Bioanalyzer High Sensitivity DNA Analysis (Agilent). Samples were pooled and sequenced on an Illumina NovaSeq platform using paired-end sequencing, with 10 million reads per sample at the Clinical Research Institute of Montreal (IRCM), Montréal, Canada.

Initial sequence quality control and adapter removal were performed using Trim Galore (v0.6.10-1). High-quality trimmed reads were mapped to the Mus musculus reference genome (mm10) using Bowtie2 (v2.5.4).^32^ Resulting SAM files were converted to BAM format and coordinate-sorted using Samtools (v1.22.1).^33^ Duplicate reads were identified and removed using Picard (v3.4.0) MarkDuplicates. Reads overlapping with ENCODE DAC blacklisted regions (ENCFF547MET)^29^ were excluded using bedtools (v2.31.1).^34^ Only high-quality (MAPQ ≥30) and properly paired (flag -f 2) alignments were kept. Reads mapping to the mitochondrial genome (chrM) were discarded. For visualization in the Integrative Genomics Viewer (IGV),^36^ RPKM-normalized bigWig files were generated using deepTools (v3.5.6)^35^ bamCoverage with a bin size of 20bp. Enrichment across annotated gene bodies was calculated using deepTools computeMatrix^38^ in scale-regions mode and subsequently visualized using plotHeatmap.

#### RNA extraction and qPCR

RNA was extracted using TRIzol reagent (15596026; Invitrogen) following the manufacturer’s protocol. RNA was normalized and used for RT-PCR followed by qPCR using EvaGreen (1725202; Bio-Rad) and the 7500 Fast Real-Time PCR system (Applied Biosystems). Transcript levels were normalized to an average to at least two housekeeping genes among *TBP, GAPDH* and *B2M* and then to the control condition (ddCT) and reported as foldchange of the ddCT.

#### CUT&RUN qPCR

Cells were harvested and subjected to CUT&RUN using the CUTANA CUT&RUN kit (14–1048; EpiCypher). In brief, cells were incubated with protein A-MNase and primary antibodies overnight at 4 °C. Targeted chromatin fragments were released by activating MNase with calcium, followed by DNA extraction. DNA concentration was quantified using PicoGreen (P7581; Invitrogen). qPCR was performed with primers (Table S1) targeting specific genomic regions of interest to assess enrichment of chromatin marks. Fold enrichment was calculated relative to the IgG control.

#### Bulk and single-cell RNA seq analysis

Public scRNA-seq data were obtained from GEO: GSE138826.^17^ Analysis was performed using the Seurat (v5.4.0) R package.^37^ Following normalization and dimensionality reduction, *Pax7*+ MuSCs were identified and sub-clustered. To visualize Notch signaling genes expression across adult myogenesis, cell-state averaged expression values for Notch pathway genes were calculated across the regeneration time course (non-injured to 21dpi). Heatmap was generated using ComplexHeatmap package,^38^ displaying *Z* score normalized expression values ordered by the difference in expression at 2dpi relative to the quiescent (non-injured) state.

Previously generated bulk RNA-seq data from WT and Kmt5a^KO^ MuSCs were reanalyzed from GEO: GSE228013. Differential expression values used were derived from the published dataset described previously.^12^

### Quantification and statistical analysis

All immunofluorescence image acquisition and quantification were performed in a double-blind manner: slides were coded prior to imaging and decoded only after all quantification was complete. Cell counting was performed on a minimum of three independent tissue sections from three separate slides per animal, and all quantifications include at least three independent biological replicates unless otherwise stated.

For CUT&Tag experiments, biological replicates consisted of independently isolated MuSC populations from separate cohorts of mice. Quiescent MuSC CUT&Tag was performed with one mouse per replicate per antibody condition (*n* = 3 independent replicates). Activated MuSC CUT&Tag was performed in triplicate across three independent experiments, each pooling MuSCs from 3 to 4 individually injured mice per antibody condition, reflecting the substantially lower cell yield from activated preparations (∼50,000 cells per injured muscle). All CUT&Tag experiments included IgG isotype controls (13–0042; EpiCypher) processed in parallel along with a positive control, H3K4me3 (Ab8580; Abcam) or H3K27me3 (9733S; Cell Signaling Technology). Unbiased peak calling and downstream genomic analyses were performed without post-hoc selection of loci.

RNA-seq differential expression analysis was performed on published data GEO: GSE228013 using the previously described pipeline,^12^ with Notch pathway enrichment identified through unbiased pathway analysis prior to the targeted validation presented here.

Statistical analyses were performed in GraphPad Prism or R. Data are shown as mean ± s.d. unless otherwise indicated. Two-group comparisons were performed using two-tailed Welch’s t tests. Multiple-group comparisons were performed using one-way ANOVA with Tukey’s multiple-comparison correction, as appropriate. All statistical tests were selected prior to analysis, and exact *p*-values are reported throughout. No data points were excluded from any analysis.

Statistical details for each experiment, including the statistical test used, are provided in the corresponding figure legends and Results where appropriate.
