# Supplementary material for: KMT5A maintains muscle stem cell quiescence through epigenetic regulation of Notch signaling
Source: iScience. 2026 Sep 4;29(9):117424. doi: 10.1016/j.isci.2026.117424 (PMC13571648; doi:10.1016/j.isci.2026.117424)
Supplement: Supplementary file 1 [file mmc1.pdf]

iScience, Volume *29*

Supplemental information

KMT5A maintains muscle stem cell quiescence through epigenetic regulation of Notch signaling

Jeonghye Hannah Hwang, Adam Y. Chu, and Roméo S. Blanc

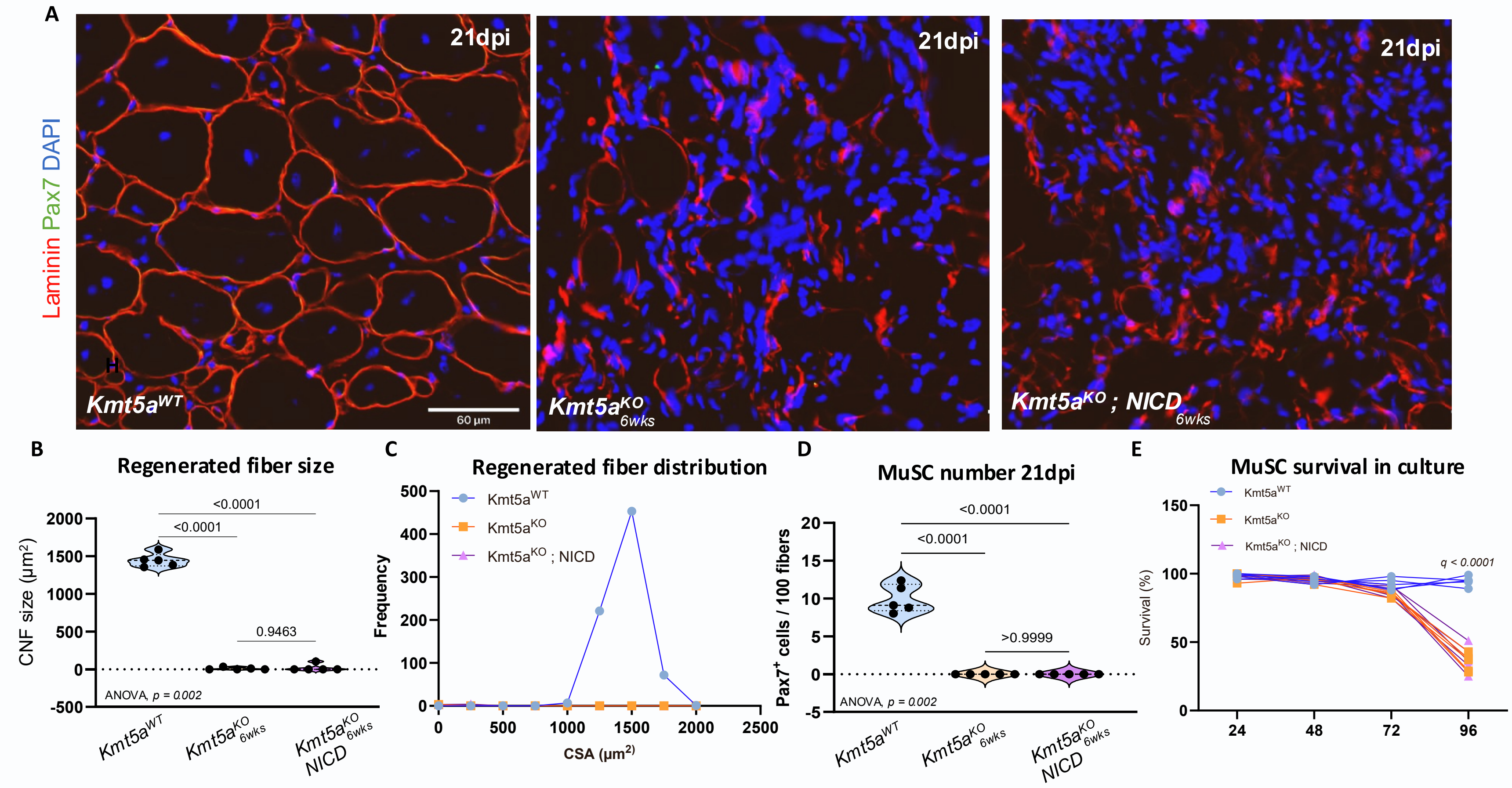

Figure S1. Enforced Notch activation does not prevent MuSC death in the absence of KMT5A, related to Figure 2.

(A, B) Representative pictures and quantification of regenerated centrally nucleated fiber (CNF) cross-sectional area (CSA). Scale bars, 60 µm. Both Kmt5a^{KO} and Kmt5a^{KO};NICD muscles exhibit a near-complete absence of regenerated fibers compared to WT controls. **(C)** Frequency distribution of regenerated fiber CSA demonstrating loss of the regeneration-associated peak (~1500 µm²) in both Kmt5a^{KO} and Kmt5a^{KO};NICD muscles. **(D)** Quantification of Pax7$^+$ MuSCs per 100 fibers at 21 dpi. Both Kmt5a^{KO} and Kmt5a^{KO};NICD muscles show near-complete depletion of the MuSC pool post-regeneration. **(E)** Ex vivo survival assay of MuSCs in culture. Viability was assessed every 24 hours. Both Kmt5a^{KO} and Kmt5a^{KO};NICD MuSCs exhibit progressive loss of survival beginning at 72 hours, whereas WT MuSCs maintain viability (n = 3 mice per genotype). Data are presented as mean ± s.d. unless otherwise indicated. Statistical significance was determined using one-way ANOVA with multiple-comparison correction, as appropriate. Exact p-values and adjusted p-values are reported in the figure.

Table S1. List of primer sequences used for qRT-PCR

**Gene**	**Forward Primer (5'-3')**	**Reverse Primer (5'-3')**
Rbpj	GGTCCCAGACATTTCTGCAT	GGAGTTGGCTCTGAGAATCG
Hes1	TGAAAGTCAAGTAAAAGGACGTAAG	AGGCTTTGATGACTTTCTGTGC
Hey1	GTTAACTCCTCCTTGCCCGC	CTCGATGATGCCTCTCCGTC
Notch1	ACAGTGCAACCCCCTGTATG	CCGCAGAAAGTGGAAGGAGT
Pax7	TACCCGGACATCTACACCCG	TACTGTGCTGCCTCCATCTTG
Myf5	GACGGCATGCCTGAATGTAAC	GCTGGACAAGCAATCCAAGC
MyoD	TGCTCTGATGGCATGATGGAT	CACTGTAGTAGGCGGTGTCG
Tbp	GGTATCTGCTGGCGGTTT	GAAATAGTGATGCTGGGCAC
Gapdh	AGGTCGGTGTGAACGGATTT	ATGAAGGGGTCGTTGATGGC
B2m	TCACACTGAATTCACCCCCA	TCACATGTCTCGATCCCAGT
Kmt5a	GCTAGAGGCAGGAAGATGTGC	CTGCCCAGCAAACACGTTCT
